# Impact of liuwei dihuang pills (decoction) on early diabetic nephropathy: a systematic review and meta-analysis

**DOI:** 10.3389/fmed.2026.1802975

**Published:** 2026-04-13

**Authors:** Haiyan Du, Huixia Ren, Naijin Zhang, Yonghui Li, Bingnan Zhang, Tingjing Ren, Hongwu Wang

**Affiliations:** College of Public Health and Health Sciences, Tianjin University of Traditional Chinese Medicine, Tianjin, China

**Keywords:** early diabetic nephropathy, liuwei dihuang decoction, liuwei dihuang pills, meta, systematic review

## Abstract

**Objective:**

To systematically evaluate the efficacy and safety of Liuwei Dihuang Pills (Decoction) combined with conventional Western medicine in the treatment of early diabetic nephropathy.

**Methods:**

Randomized controlled trials (RCTs) on Liuwei Dihuang Pills (Decoction) combined with conventional treatment for early diabetic nephropathy were retrieved from major domestic and foreign databases from their inception to December 2025. Two researchers independently conducted literature screening, data extraction, and bias risk assessment. Meta-analysis was performed using Reviewer Manager 5.4 software. The primary outcome indicator was urinary albumin excretion rate (UAER); secondary outcomes included renal function indicators, total effective rate, and adverse events.

**Results:**

A total of 11 RCTs involving 1,007 patients were included. Meta-analysis results showed that, compared with conventional Western medicine alone, the combination group significantly reduced UAER (MD = −37.30, 95% CI: −47.51 to −27.09, *P* < 0.00001), with substantial heterogeneity (I^2^ = 93%); sensitivity analysis confirmed the robustness of the result after excluding one study. Renal function indicators also improved significantly: serum creatinine (MD = −5.96, 95% CI: −9.27 to −2.65, *P* = 0.0004) and blood urea nitrogen (MD = −0.91, 95% CI: −1.46 to −0.36, *P* = 0.001). The total effective rate was higher in the experimental group (OR = 5.10, 95% CI: 3.38 to 7.68, *P* < 0.00001), though this outcome should be interpreted with caution due to the lack of blinding and the subjective nature of the indicator. Adverse events were reported in only 6 of the 11 studies; the experimental group had 12/287 (4.2%) cases and the control group had 12/277 (4.3%) cases, with most reported events being mild to moderate. The pooled analysis showed no significant difference between groups (OR = 0.99, 95% CI: 0.43 to 2.30, *P* = 0.99), and most reported events were mild to moderate. However, incomplete safety reporting limits the reliability of this finding.

**Conclusion:**

Liuwei Dihuang Pills (Decoction) combined with conventional Western medicine may provide additional benefits for patients with early diabetic nephropathy, showing potential advantages in improving objective renal function indicators and clinical outcomes. The available safety data suggest no significant increase in adverse events, but this finding is limited by incomplete reporting across the included studies. Due to the limited sample size, insufficient methodological quality (including lack of allocation concealment and blinding), and high heterogeneity in some analyses, and incomplete adverse event reporting, the overall evidence quality is low. High-quality, large-sample, multi-center RCTs with rigorous methodological design and standardized safety reporting are still needed to further verify the above conclusions.

**Systematic review registration:**

https://www.crd.york.ac.uk/PROSPERO/view/CRD420261280431, identifier: CRD420261280431.

## 1 Introduction

Diabetic nephropathy is one of the severe microvascular complications of diabetes mellitus (1), and also a leading cause of renal failure in diabetic patients (2). With the rapid development of the economy and the improvement of people's living standards, the prevalence of diabetes is on the rise. It is estimated that by 2023, the global prevalence of diabetes will reach 463 million people (accounting for 9.3% of adults aged 20–79), and this number is expected to rise to 700 million by 2045 (3). Epidemiological studies have shown that approximately 35%−40% of patients with diabetes mellitus may progress to diabetes-related nephropathy (4), and this disease has become a major cause of end-stage renal disease (ESRD) in many countries worldwide (5), which it is increasingly emerging as a significant health and socioeconomic issue (6). Diabetic nephropathy has an insidious onset and is characterized by microalbuminuria at the early stage, during which renal injury is still potentially reversible (7). Delayed intervention often leads to irreversible renal function decline. Therefore, targeted the early prevention of the occurrence and progression of diabetic nephropathy (DN) is of great significance for improving patients' quality of life and alleviating the national medical burden (8). In the field of traditional Chinese medicine (TCM), diabetic nephropathy falls into the categories of “Xiaoke” (Wasting-Thirst) and “Shuizhong” (Edema) (9). Its pathogenesis is mainly characterized by qi deficiency of the spleen and kidney, kidney yin deficiency, internal accumulation of dampness-heat, and qi stagnation and blood stasis (10). Liuwei Dihuang Decoction is a core formula for nourishing yin, with its main effects including nourishing the liver and kidney, and also replenishing the spleen yin—it is thus recognized as a formula that nourishes the “three yin” (liver yin, kidney yin, and spleen yin) simultaneously (11). It has demonstrated certain advantages in the treatment and prevention of early diabetic nephropathy, as well as in improving the clinical symptoms of diabetic nephropathy.

The formula of Liuwei Dihuang Decoction consists of six Chinese herbs: Rehmannia (prepared), Cornus officinalis, Moutan, Chinese yam, Poria, and Alisma. The main active compound in Rehmannia and Cornus officinalis significantly improves renal function in the db/db diabetic mouse model by inhibiting the AGEs/RAGE/SphK1 signaling pathway. This results in reduced proteinuria, serum creatinine, and urea nitrogen levels, as well as alleviation of glomerular basement membrane thickening, mesangial matrix expansion, and renal fibrosis. Additionally, it improves pathological damage to the pancreas and kidneys (12). The terpenoid compounds in Moutan Cortex inhibit endoplasmic reticulum stress-related inflammatory responses. In diabetic nephropathy rat models and AGEs-induced mesangial cells, these compounds significantly improve renal function and pathological damage, reducing the expression of inflammatory markers such as IL-6, MCP-1, and ICAM-1 (13). The water extract of Chinese yam reduces serum urea nitrogen, creatinine, and uric acid levels in an acetaminophen-induced acute kidney injury rat model, improving renal tubular degranulation, necrosis, and collapse (14). Alisma's main active compound, alisol B, alleviates apoptosis, inflammation, and oxidative stress in cisplatin-induced acute kidney injury by inhibiting soluble epoxide hydrolase (sEH) and regulating the GSK3β-mediated p53, NF-κB, and Nrf2 signaling pathways (15). Poria and its active compounds protect kidney function through multiple mechanisms. Poricoic acid A primarily inhibits the ubiquitination degradation of SPRY2, upregulating its expression, and blocking the phosphorylation activation of the ERK signaling pathway (16). This significantly suppresses TGF-β1-induced epithelial-mesenchymal transition (EMT) in renal tubular epithelial cells, reducing extracellular matrix deposition, cell migration, and invasion. Furthermore, Poria protects renal function by inhibiting the renal RAS system, blocking TGF-β/Smad and Wnt/β-catenin fibrosis pathways, activating the Nrf2 antioxidant pathway, and suppressing NF-κB inflammatory signaling (17). Its water-soluble polysaccharides also alleviate inflammation, oxidative stress, and apoptosis in sepsis-induced acute kidney injury through inhibition of the NF-κB-NOX4 signaling axis (18). Additionally, its Chinese patent medicine form, Liuwei Dihuang Pills, is economically affordable and particularly popular among most patients in rural primary care settings in China. Thus, it demonstrates great potential for clinical application.

Existing systematic reviews have all focused on diabetic nephropathy as a whole, with no systematic review specifically targeting early diabetic nephropathy. The present study intends to evaluate the efficacy and safety of Liuwei Dihuang Pills (Decoction) combined with conventional Western medicine in the treatment of early diabetic nephropathy through a Meta-analysis. It aims to provide a reference for clinicians in the management and treatment of early diabetic nephropathy, and the details are reported as follows.

## 2 Method

### 2.1 Protocol and registration

This systematic review and meta-analysis was conducted in accordance with the Preferred Reporting Items for Systematic review and Meta-Analysis (PRISMA) extension guidelines (19). It was preregistered in the International Prospective Register of Systematic Reviews (PROSPERO) (20) (registration: CRD420261280431).

### 2.2 Search strategy

Primary studies published publicly domestic and international were retrieved from the EMBASE, PubMed, The Cochrane Central Register of Controlled Trials (CENTRAL), Web of Science, China National Knowledge Infrastructure (CNKI), Chinese Science and Technology Journal Database (VIP), and Wanfang Data Knowledge Service Platform. The retrieval period was from the establishment of each database to January 2026. For the retrieval process “早期糖尿病肾病,” “糖尿病肾病早期,” “2 型糖尿病肾病早期,” “糖尿病合并肾病早期,” “六味地黄丸,” “六味地黄汤,” “early diabetic kidney disease,” “diabetic early ne-phropathy,” “Liuweidihuang Pill,” “liu-wei-di-huang wan,” “ Liuwei Dihuang Decoction,” and other search terms. The Chinese retrieval methods are as follows: (主题:早期糖尿病肾病) OR [篇关摘:糖尿病肾病早期 (精确)] OR [篇关摘:2型糖尿病肾病早期 (精确)] OR [篇关摘:糖尿病合并肾病早期 (精确)] AND (主题:六味地黄丸) OR [篇关摘:六味地黄汤 (精确)] OR [篇关摘:六味地黄 (精确)]; The English retrieval method is as follows:(early diabetic kidney disease OR diabetic early nephropathy OR early-stage diabetic nephropathy OR early diabetic nephropathy) AND (liuwei dihuang pill OR liuwei dihuang pills OR liu-wei-di-huang wan OR liuwei dihuang wan OR Liuwei Dihuang Decoction). The Endnote X9 software was used to collect all literature related to the above search terms from Chinese and English databases.

### 2.3 Study selection

(1) Inclusion criteria: ① Study type: publicly published randomized controlled trials (RCTs) on the treatment of early diabetic nephropathy with Liuwei Dihuang Pills (Decoction), with the literature language being Chinese or English; ② Study subjects: patients who meet the diagnostic criteria for early diabetic nephropathy, regardless of gender, age, severity of illness, course of disease, ethnicity, etc.; ③ Intervention measures: the control group receives conventional western medicine treatment, and the experimental group receives Liuwei Dihuang Pills (Decoction) in addition to conventional treatment; there is no restriction on the dosage and course of treatment for both groups; ④ Outcome indicators: The primary outcome indicator was urinary albumin excretion rate (UAER), which is a core objective parameter for the diagnosis and efficacy evaluation of early diabetic nephropathy. Secondary outcome indicators included serum creatinine (Scr), blood urea nitrogen (BUN), mean arterial pressure, urinary microalbumin, total effective rate, and adverse events. The total effective rate was calculated as: total effective rate = (number of markedly effective cases + number of effective cases) / total cases in each group × 100%. It should be noted that the criteria for “markedly effective” and “effective” varied across the included studies, and this indicator is based on the improvement of clinical symptoms, which is susceptible to subjective factors and placebo effects given the lack of blinding in all included studies. (2) Exclusion criteria: ① Non-RCT studies; ② Duplicate published literature; ③ Literature with invalid data or inappropriate statistical methods; ④ Literature where the original text cannot be viewed, such as animal experimental studies, case reports, reviews, etc.; ⑤ Literature where the intervention measures involve combining with other traditional Chinese medicines or modified Liuwei Dihuang Pills (Decoction).

The retrieved literatures were imported into EndnoteX9. After removing duplicate literatures, two researchers (DHY, RHX) conducted preliminary screening, respectively. Those that met the criteria were further read in full text and data were extracted. Finally, cross-checking was performed to determine whether to include them. If there was any disagreement, it would be discussed by the two researchers (DHY, RHX) or adjudicated by a third party (ZNJ).

### 2.4 Data extraction

A standardized form was used to extract data from the eligible studies. The extracted information included the following: first author, year of publication, sample size, age, gender, intervention measures, treatment course, and outcome indicators. Two authors (LYH and ZBN) cross-checked the accuracy of the data extracted from each study. An independent third author (DHY) resolved all inconsistencies.

### 2.5 Risk of bias assessment

The assessment was conducted in accordance with the quality evaluation criteria for randomized controlled trials in the Cochrane Handbook for Systematic Reviews. The specific evaluation criteria include: ① Description of the random allocation method and its scientific verification (low risk: use of reliable methods such as random number tables, computer-generated random sequences, etc.; high risk: use of non-scientific methods such as patient age, parity of outpatient numbers, parity of inpatient numbers, etc.; unclear risk: failure to elaborate on the method, making it difficult to evaluate); ② Adequacy of the allocation concealment scheme (low risk: implementation of central randomization; high risk: random allocation sequence is public, with a risk of disclosure; unclear risk: no mention of allocation concealment measures); ③ Use of blinding (low risk: although blinding was not adopted, the outcome assessment was not affected; high risk: blinding was not implemented and the outcome assessment was affected; unclear risk: no mention of the application of blinding); ④ Completeness of outcome data (low risk: missing data do not affect result analysis or there is no missing data; high risk: missing data have a significant impact on result analysis; unclear risk: there is missing information, but it is difficult to assess the impact); ⑤ Selective reporting of research results (low risk: there is a preset research plan and results are reported in accordance with the plan, or there is no plan but outcome indicators are comprehensive; high risk: failure to report preset primary outcome indicators; unclear risk: no specific elaboration); ⑥ Other potential sources of bias (low risk: no other bias factors found; high risk: such as early termination of the study, etc.; unclear risk: no specific explanation).

### 2.6 Data analysis

Meta-analysis was performed using Reviewer Manager 5.4: binary variables were expressed as relative risk (RR) and 95% confidence interval (CI); continuous data were expressed as mean difference (MD) and 95% CI. Heterogeneity among studies was analyzed by Q test. If there was no significant statistical heterogeneity among studies in the group (when *P* > 0.1, I^2^ < 50%), a fixed-effects model was used for analysis; if there was heterogeneity (when *P* < 0.1, I^2^ > 50%), a random-effects model was used for analysis (21). Meanwhile, a funnel plot was generated to analyze the publication bias of the included studies.

## 3 Results

### 3.1 Literature retrieval results

**Figure 1:**
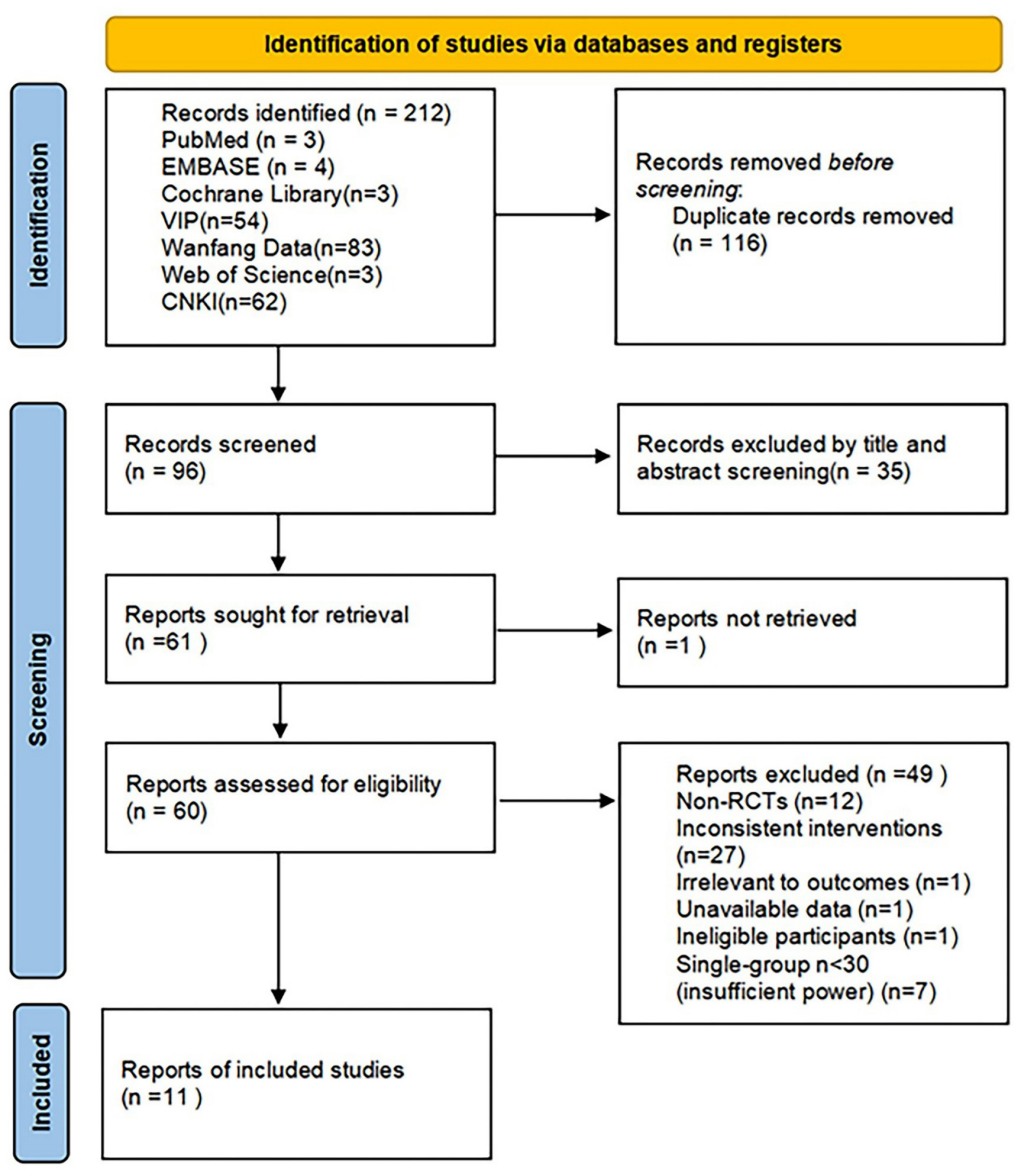
PRISMA flow diagram of study selection for the systematic review and meta-analysis of Liuwei Dihuang Pills (Decoction) combined with conventional Western medicine in the treatment of early diabetic nephropathy. The diagram illustrates the complete process of literature retrieval, screening, and inclusion: initial retrieval of 212 records from seven databases (PubMed, EMBASE, Cochrane Library, Web of Science, CNKI, Wanfang Data, VIP); removal of 116 duplicate records; title and abstract screening to exclude 35 irrelevant records; exclusion of 1 record that could not be retrieved; full-text assessment of 60 remaining records, with 49 excluded for reasons including non-randomized controlled trials (*n* = 12), inconsistent interventions (*n* = 27), irrelevant outcomes (*n* = 1), unavailable data (*n* = 1), ineligible participants (*n* = 1), and insufficient sample size (single group *n* < 30, *n* = 7); finally, 11 eligible randomized controlled trials (RCTs) were included in the meta-analysis.

A preliminary search retrieved 212 relevant articles from seven databases: PubMed contributed 3 records, EMBASE contributed 4 records, Cochrane Library contributed 3 records, Web of Science contributed 3 records, CNKI contributed 62 records, Wanfang Data contributed 83 records, and VIP contributed 54 records. After importing them into EndnoteX9 software, 116 duplicate articles were excluded, leaving 96 articles. After further step-by-step screening, 35 articles that did not match the research content were excluded by reading their titles and abstracts, followed by excluding 1 article that could not be obtained. After carefully reading the remaining 60 articles, 49 were excluded for the following reasons: 12 articles were non-randomized controlled trials, 27 articles had inconsistent interventions, 1 article was irrelevant to the outcomes, 1 article had unavailable data, 1 article involved ineligible participants, and 7 articles had insufficient sample size with fewer than 30 participants per group. Finally, 11 Chinese articles on RCT studies that met the criteria were finally included. The process and results of literature screening are shown in Figure 1.

### 3.2 Basic information and quality evaluation of included studies

A total of 11 included RCTs involved 1,007 patients with early diabetic nephropathy, including 508 cases in the experimental group and 489 cases in the control group. All included literatures clearly stated the baseline conditions of the patients. 10 studies (22–31) reported the total effective rate;4 studies(22, 23, 27, 28) reported serum creatinine; 4 studies (22, 23, 27, 28) reported blood urea nitrogen; 3 studies(27, 28, 30) reported the mean arterial pressure; 4 studies(27, 28, 30, 32) reported the urinary albumin excretion rate; 3 studies(24, 27, 29) reported urinary microalbumin; 6 studies(22, 23, 27–29, 32) reported adverse reactions. The basic characteristics of the included literature are detailed in Table 1.

**Table 1:** Basic information included in the study.

Study (author, year)	Sample size	Age (years)	Sex (Male/female)	Intervention (T)	Dosing time (week)	Main outcomes
Chen et al. (23)	T:44 C:43	T:58.62 ± 5.12 C:58.6 ± 5.08	T:23/21 C:23/20	A + Liuwei Dihuang Pills	8	①⑦③②
Li (22)	T:49 C:49	T:58.6 ± 5.02 C:58.65 ± 4.78	T:27/22 C:26/23	A + Liuwei Dihuang Pills	8	①③②⑦
Zhang et al. (24)	T:40 C:40	T:45.77 ± 14.05 C:44.56 ± 13.27	T:22/18 C:25/15	A+ Liuwei Dihuang Decoction	4	①⑥
Cui (32)	T:49 C:49	T:50.1 ± 7.4 C:50.8 ± 6.5	T:26/23 C:24/25	A + Liuwei Dihuang Pills	12	⑤
Guo et al. (25)	T:50 C:50	T:65.41 ± 1.17 C:66.28 ± 1.26	T:29/21 C:31/19	A + Liuwei Dihuang Pills	4	①
Wang (27)	T:50 C:50	T:65.24 ± 8.63 C:64.96 ± 7.65	T:26/24 C:22/28	A + Liuwei Dihuang Pills	8	①④②③⑥⑤⑦
Ma (28)	T:74 C:74	T:57.0 ± 5.8 C:54.0 ± 5.8	T:39/35 C:30/44	A + Liuwei Dihuang Pills	24	①②③⑤④⑦
Wan (26)	T:37 C:37	T:69 C:67	T:18/19 C:19/18	A + Liuwei Dihuang Pills	24	①
Cao (29)	T:36 C:36	T:52.1 ± 3.7 C:51.7 ± 3.2	T:22/14 C:20/16	A + Liuwei Dihuang Pills	8	①⑥⑦
Song et al. (30)	T:41 C:31	T:39.9 ± 17.5 C:40.7 ± 19.9	T:28/13 C:11/20	A + Liuwei Dihuang Pills	12	①④⑤
Chen et al. (31)	T:38 C:30	56.4 ± 5.2	30/38	A + Liuwei Dihuang Pills	12	①

T, experimental group; C, control group; A, conventional treatment, including lifestyle modifications, blood glucose control, blood pressure reduction, lipid regulation, and other symptomatic treatments; the intervention measures for the control group are all conventional treatment; ① total effective rate; ② Scr; ③ BUN; ④ mean arterial pressure; ⑤ urinary albumin excretion rate; ⑥ urinary microalbumin; ⑦ adverse reactions.

### 3.3 Quality assessment of included studies

**Figure 2:**
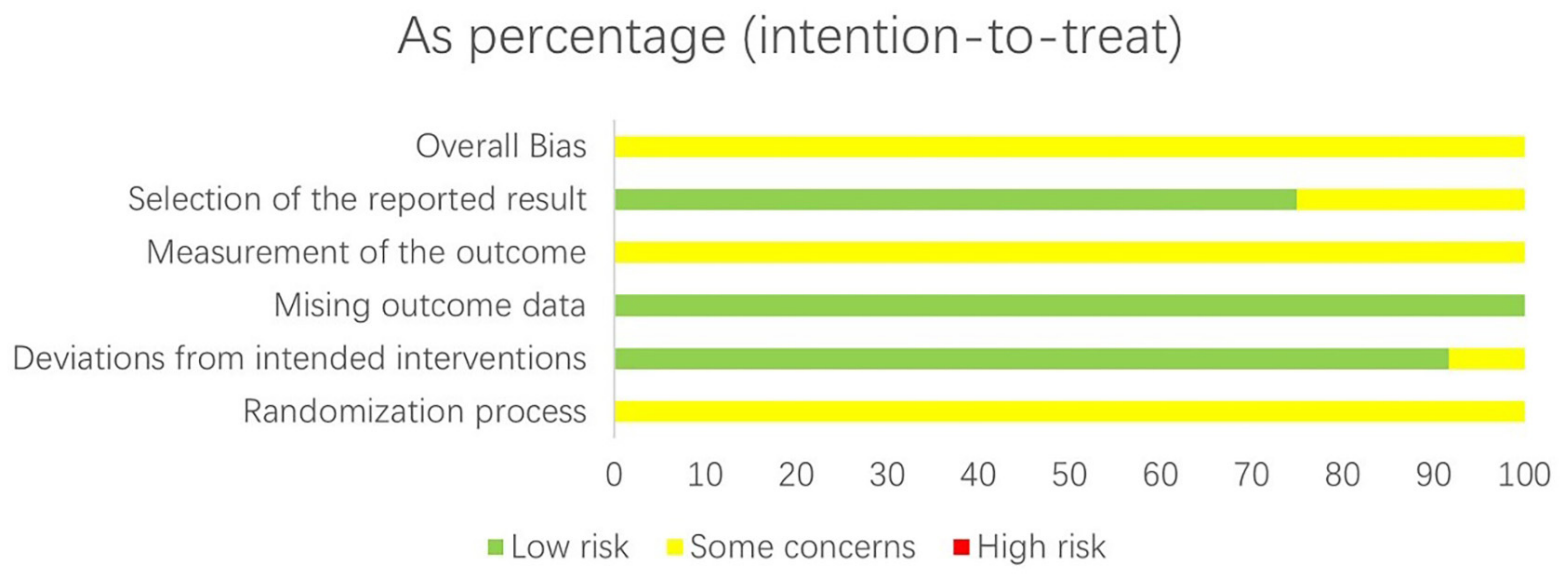
Risk of bias summary plot of the 11 included RCTs assessed by the Cochrane Risk of Bias Tool 2.0. Each row represents a key bias assessment domain (randomization process, deviations from intended interventions, missing outcome data, measurement of the outcome, selection of the reported result, overall bias), and each column represents an individual included study. The color coding indicates the review authors' judgements for each domain: green for low risk of bias, yellow for some concerns, and red for high risk of bias. All assessments were conducted independently by two researchers, with discrepancies resolved by a third independent reviewer.

**Figure 3:**
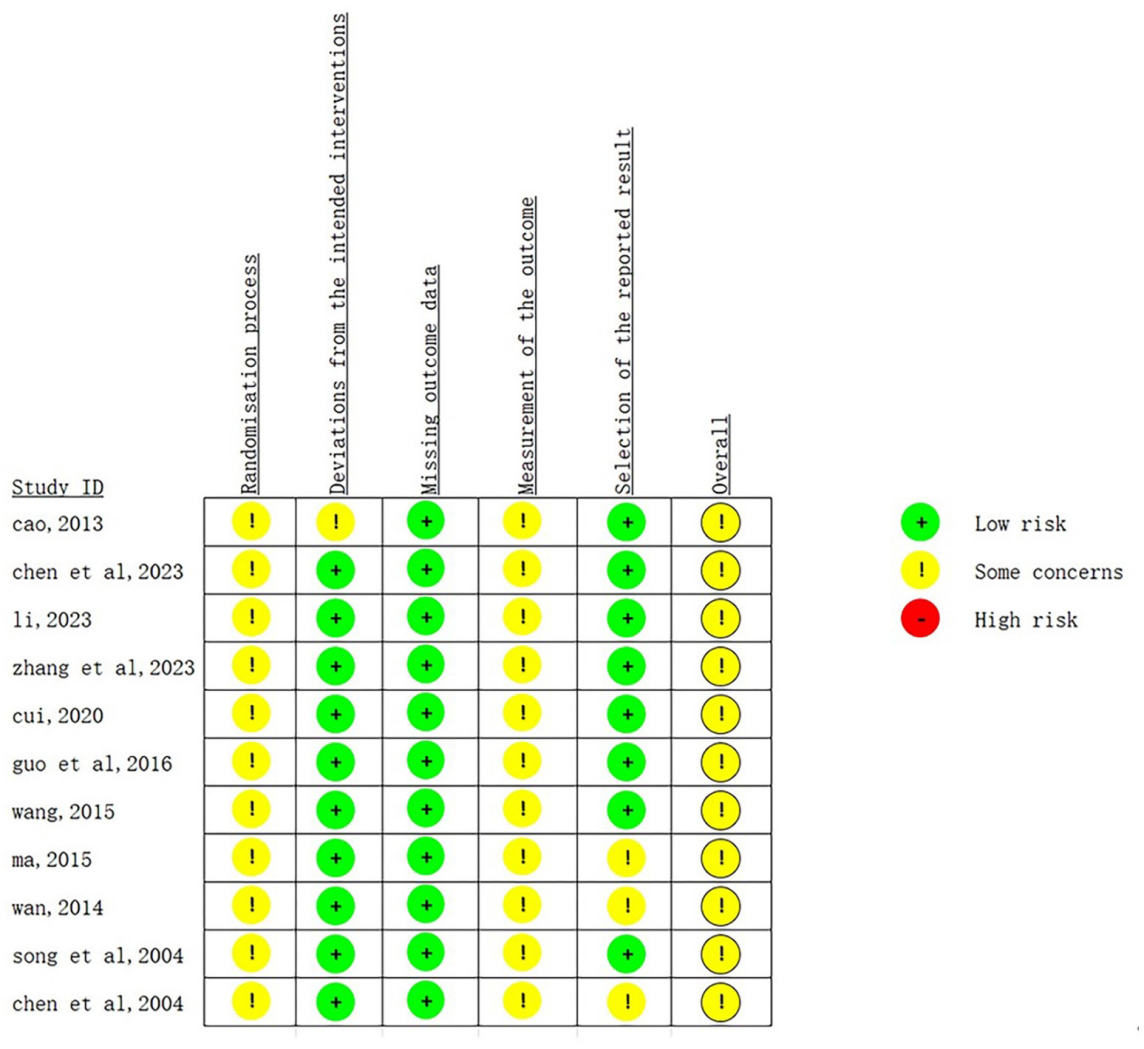
Risk of bias graph of the 11 included RCTs assessed by the Cochrane Risk of Bias Tool 2.0. The graph displays the proportion of included studies rated as low risk, some concerns, or high risk of bias for each core assessment domain (randomization process, deviations from intended interventions, missing outcome data, measurement of the outcome, selection of the reported result). The x-axis represents the percentage of studies, and the y-axis lists the bias assessment domains; different colors distinguish the risk of bias levels (low, some concerns, high).

A total of 11 literatures were included in this study and evaluated according to the Cochrane Handbook for Systematic Reviews: (1) All 11 studies mentioned random allocation, 5 studies mentioned the use of random number table method for allocation, 1 study used the lottery method, and the rest only mentioned “random”; (2) None of the included studies reported the allocation concealment scheme; (3) None of the included studies mentioned blinding; (4) None of the included studies had loss to follow-up; (5) None of the included studies reported sample size estimation. The quality assessment of the included studies is shown in Figures 2, 3.

### 3.4 Efficacy analysis of included studies

#### 3.4.1 Urinary albumin excretion rate

**Figure 4:**
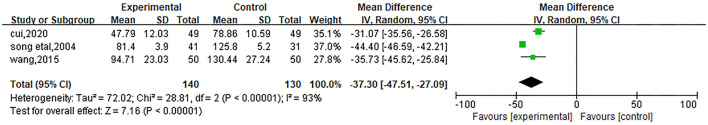
Forest plot of the effect of Liuwei Dihuang Pills (Decoction) combined with conventional Western medicine on urinary albumin excretion rate (UAER, μg/min) in patients with early diabetic nephropathy. The plot includes 3 eligible RCTs (27, 30, 32) with a total of 140 patients in the experimental group and 130 in the control group. UAER units were unified by converting mg·24 h^−1^ to μg/min (1 mg·24 h^−1^ = 0.694 μg/min) for consistency. A random-effects model was used due to high heterogeneity (I^2^ = 93%, *P* < 0.00001). Each horizontal line represents the 95% confidence interval (CI) of the mean difference (MD) for an individual study, and the black square represents the weighted MD; the diamond at the bottom represents the pooled MD and 95% CI for all included studies (MD = −37.30, 95% CI: −47.51 to −27.09, *P* < 0.00001). The left side of the vertical dashed line (null effect) indicates a favorable effect of the experimental intervention.

Included 3 studies (27, 30, 32). Among them, the units of measurement exist in two forms: mg·24 h^−1^ and μg/min. To ensure the comparability of data, before the Meta-analysis, the relevant data were processed to unify the units based on the conversion relationship of 1mg·24 h^−1^ = 0.694 μg/min. After unified conversion to μg/min, the mean difference (MD) and 95% confidence interval (CI) were used for pooled analysis. Due to high heterogeneity (*P* < 0.00001, I^2^ = 93%), a random-effects model was used for the analysis. The meta-analysis showed that the MD value of the total effect was −37.30, with a 95% confidence interval (95%CI) of [−47.51, −27.09], and *P* < 0.00001, indicating that the urinary albumin excretion rate (μg/min) index in the experimental group after treatment was significantly lower than that in the control group. The results are shown in Figure 4.

**Figure 5:**
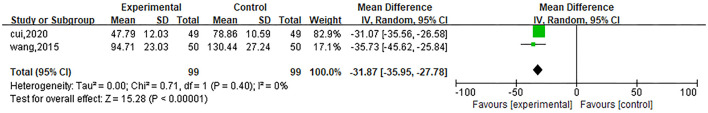
Sensitivity analysis forest plot of UAER (μg/min) after excluding the study by Song et al. (30) (the main source of heterogeneity). The plot includes 2 remaining RCTs (27, 32) with 99 patients in both the experimental and control groups. A random-effects model was applied, and heterogeneity was substantially reduced to I^2^ = 0% (*P* = 0.40). The pooled mean difference (MD = −31.87, 95% CI: −35.95 to −27.78, *P* < 0.00001) remained statistically significant, confirming the robustness of the primary UAER analysis result. Plot elements follow the same definition as Figure 4: horizontal lines = 95% CI, black squares = weighted MD, diamond = pooled effect size.

To address the high heterogeneity observed in UAER, a sensitivity analysis was performed by sequentially excluding individual studies. After excluding Song et al. (30), heterogeneity decreased substantially from 93% to 0%, and the pooled effect remained significant (MD = −31.87, 95% CI: −35.95 to −27.78, *P* < 0.00001). This suggests that the study by Song et al. (30) was the main source of heterogeneity, and the overall conclusion regarding the effect on UAER is robust. The results are shown in Figure 5.

**Figure 6:**
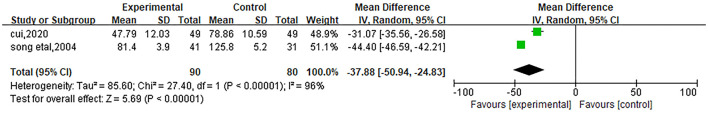
Sensitivity analysis forest plot of UAER (μg/min) after excluding the study requiring unit conversion. The plot includes 2 RCTs (30, 32) with 90 patients in the experimental group and 80 in the control group. A random-effects model was used (I^2^ = 96%, *P* < 0.00001), and the pooled mean difference (MD = −37.88, 95% CI: −50.94 to −24.83, *P* < 0.00001) remained statistically significant. This result verifies that unit conversion of UAER did not substantially affect the overall meta-analysis conclusion. Plot elements follow the same definition as Figure 4.

Additionally, to assess the potential impact of unit conversion, a sensitivity analysis was conducted excluding the study requiring unit conversion (27). The pooled effect remained consistent (MD = −37.88, 95% CI: −50.94 to −24.83, *P* < 0.00001), indicating that the unit conversion did not substantially affect the results. The results are shown in Figure 6.

#### 3.4.2 Scr

**Figure 7:**
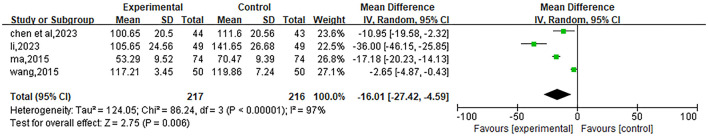
Forest plot of the effect of Liuwei Dihuang Pills (Decoction) combined with conventional Western medicine on serum creatinine (Scr, μmol/L) in patients with early diabetic nephropathy. The plot includes 4 eligible RCTs (22, 23, 27, 28) with 217 patients in the experimental group and 216 in the control group. A random-effects model was used due to extreme heterogeneity (I^2^ = 97%, *P* < 0.00001). The pooled mean difference (MD = −16.01, 95% CI: −27.42 to −4.59, *P* = 0.006) indicates a statistically significant reduction in Scr in the experimental group compared with the control group. Plot elements follow the same definition as Figure 4.

Included 4 studies (22, 23, 27, 28). Due to significant heterogeneity (*P* < 0.00001, I^2^ = 97%), a random-effects model was used for the analysis. The meta-analysis showed that the MD value of the total effect was −16.01, with a 95% confidence interval (95%CI) of [−27.42, −4.59], and *P* = 0.006, indicating that the mean value of the Scr (μmol/L) index in the experimental group was significantly lower than that in the control group. The above outcomes have high heterogeneity, which may be related to differences in treatment courses, basic medications, and included populations, and the results need to be interpreted with caution. The results are shown in Figure 7.

**Figure 8:**
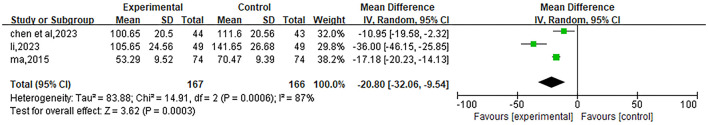
Sensitivity analysis forest plot of Scr (μmol/L) after excluding the study by Wang (27) (the main source of heterogeneity). The plot includes 3 remaining RCTs (22, 23, 28) with 167 patients in the experimental group and 166 in the control group. A random-effects model was applied, with heterogeneity reduced to I^2^ = 87% (*P* = 0.0006). The pooled mean difference (MD = −20.80, 95% CI: −32.06 to −9.54, *P* = 0.0003) remained statistically significant, indicating the stability of the Scr analysis result despite persistent heterogeneity. Plot elements follow the same definition as Figure 4.

A sensitivity analysis revealed that Wang et al. (27) was the primary source of heterogeneity. After excluding this study, heterogeneity decreased to 87%, and the pooled effect remained significant (MD = −20.80, 95% CI: −32.06 to −9.54, *P* = 0.0003), consistent with the original analysis. Given the persistently high heterogeneity and the limited number of included studies, subgroup analysis could not be performed. Therefore, the results should be interpreted with caution. The results are shown in Figure 8.

#### 3.4.3 BUN

**Figure 9:**
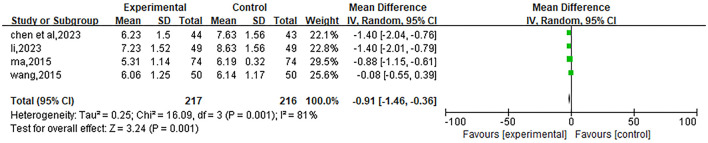
Forest plot of the effect of Liuwei Dihuang Pills (Decoction) combined with conventional Western medicine on blood urea nitrogen (BUN, mmol/L) in patients with early diabetic nephropathy. The plot includes 4 eligible RCTs (22, 23, 27, 28) with 217 patients in the experimental group and 216 in the control group. A random-effects model was used due to high heterogeneity (I^2^ = 81%, *P* = 0.001). The pooled mean difference (MD = −0.91, 95% CI: −1.46 to −0.36, *P* = 0.001) indicates a statistically significant reduction in BUN in the experimental group compared with the control group. Plot elements follow the same definition as Figure 4.

Included 4 studies (22, 23, 27, 28). Due to high heterogeneity (*P* = 0.001, I^2^ = 81%), a random-effects model was used for analysis. The meta-analysis showed that the MD value of the total effect was −0.91, with a 95% confidence interval (95%CI) of [−1.46, −0.36], and *P* = 0.001, indicating that the BUN (mmol/L) index in the experimental group after treatment was significantly lower than that in the control group. The results are shown in Figure 9.

#### 3.4.4 Mean arterial pressure

**Figure 10:**
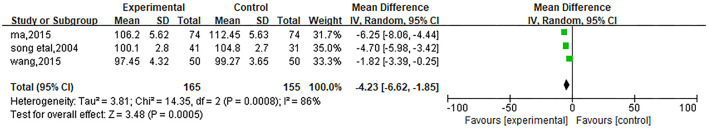
Forest plot of the effect of Liuwei Dihuang Pills (Decoction) combined with conventional Western medicine on mean arterial pressure (mmHg) in patients with early diabetic nephropathy. The plot includes 3 eligible RCTs (27, 28, 30) with 165 patients in the experimental group and 155 in the control group. A random-effects model was used due to high heterogeneity (I^2^ = 86%, *P* = 0.0008). The pooled mean difference (MD = −4.23, 95% CI: −6.62 to −1.85, *P* = 0.0005) indicates a statistically significant reduction in mean arterial pressure in the experimental group compared with the control group. Plot elements follow the same definition as Figure 4.

Included 3 studies (27, 28, 30). Due to significant heterogeneity (*P* = 0.0008, I^2^ = 86%), a random-effects model was used for the analysis. The meta-analysis showed that the MD value of the total effect was −4.23, with a 95% confidence interval (95%CI) of [−6.62, −1.85], and *P* = 0.0005, indicating that the mean arterial pressure index in the experimental group after treatment was significantly lower than that in the control group. The results are shown in Figure 10.

#### 3.4.5 Total effective rate

**Figure 11:**
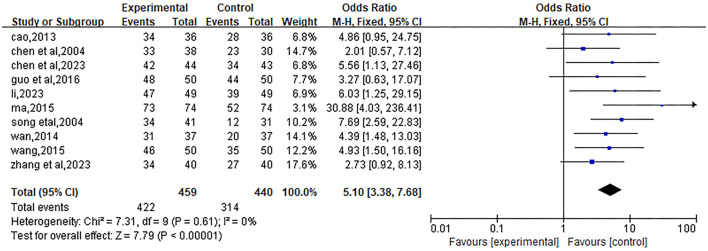
Forest plot of the effect of Liuwei Dihuang Pills (Decoction) combined with conventional Western medicine on the total effective rate in patients with early diabetic nephropathy. The plot includes 10 eligible RCTs with 459 patients in the experimental group and 440 in the control group. The total effective rate was defined as (markedly effective cases + effective cases)/total cases × 100% (criteria for marked/effective varied by study). A fixed-effects model was used due to low heterogeneity (I^2^ = 0%, *P* = 0.61). The plot reports odds ratios (OR) with 95% CIs; each horizontal line represents the 95% CI of the OR for an individual study, the black square represents the weighted OR, and the diamond represents the pooled OR (OR = 5.10, 95% CI: 3.38 to 7.68, *P* < 0.00001). The left side of the vertical dashed line (null effect, OR = 1) indicates a higher total effective rate in the experimental group.

Included 10 studies (22–31). Due to low heterogeneity (*P* = 0.61, I^2^ = 0), a fixed-effects model was used for analysis. The meta-analysis showed that the OR value of the total effect was 5.10, with a 95% confidence interval (95%CI) of [3.38, 7.68], and *P* < 0.00001, indicating that the total effective rate of the experimental group was significantly better than that of the control group. The results are shown in Figure 11.

#### 3.4.6 Microalbuminuria

**Figure 12:**
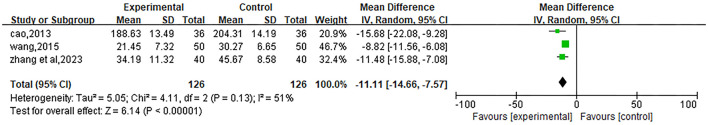
Forest plot of the effect of Liuwei Dihuang Pills (Decoction) combined with conventional Western medicine on urinary microalbumin (mg/L) in patients with early diabetic nephropathy. The plot includes 3 eligible RCTs (24, 27, 29) with 126 patients in both the experimental and control groups. A random-effects model was used due to moderate heterogeneity (I^2^ = 51%, *P* = 0.13). The pooled mean difference (MD = −1.11, 95% CI: −14.66 to −7.57, *P* < 0.00001) indicates a statistically significant reduction in urinary microalbumin in the experimental group compared with the control group. Plot elements follow the same definition as Figure 4.

Included 3 studies (24, 27, 29). Due to significant heterogeneity (*P* = 0.13, I^2^ = 51%), a random-effects model was used for analysis. The meta-analysis showed that the MD value of the total effect was −11.11, with a 95% confidence interval (95%CI) of [−14.66, −7.57], and *P* < 0.00001, indicating that the urinary microalbumin (mg/L) index in the experimental group after treatment was significantly lower than that in the control group. The results are shown in Figure 12.

#### 3.4.7 Adverse reactions

**Figure 13:**
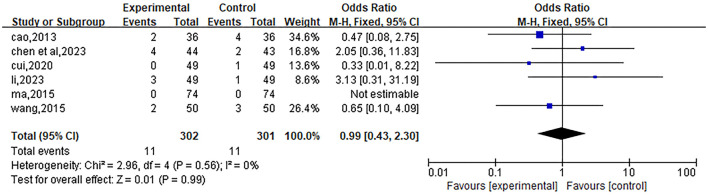
Forest plot of the effect of Liuwei Dihuang Pills (Decoction) combined with conventional Western medicine on the incidence of adverse reactions in patients with early diabetic nephropathy. The plot includes 6 eligible RCTs with 302 patients in the experimental group and 301 in the control group. Adverse reactions included fatigue, nausea, skin rashes, hypotension, headache, and gastrointestinal symptoms (all mild to moderate). A fixed-effects model was used due to no heterogeneity (I^2^ = 0%, *P* = 0.56). The plot reports odds ratios (OR) with 95% CIs (pooled OR = 0.99, 95% CI: 0.43 to 2.30, *P* = 0.99), with plot elements following the same definition as Figure 11. The vertical dashed line (OR = 1) indicates no significant difference in adverse reaction incidence between the two groups.

Included 6 studies (22, 23, 27–29, 32). Due to the small heterogeneity (*P* = 0.56, I^2^ = 0), a fixed-effects model was used for the analysis. The meta-analysis showed that the OR value of the total effect was 0.99, with a 95% confidence interval (95%CI) of [0.43, 2.30], and *P* = 0.99, indicating that there was no significant difference in the risk of adverse reactions between the experimental group and the control group after treatment. The results are shown in Figure 13.

### 3.5 Analysis of publication bias

**Figure 14:**
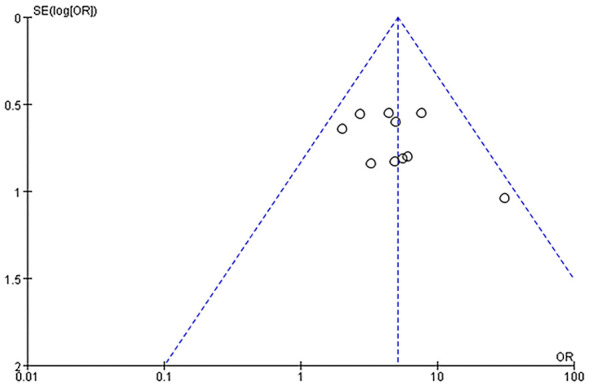
Funnel plot to assess publication bias for the total effective rate outcome. The x-axis represents the odds ratio (OR) of the total effective rate for individual studies, and the y-axis represents the standard error of the natural logarithm of the OR [SE(log(OR))]. Each dot represents an individual included study; the symmetric funnel outline represents the expected distribution of studies in the absence of publication bias. Most study points are concentrated in the right area (OR > 1) and show asymmetry toward the upper half of the funnel, suggesting potential publication bias (likely underreporting of studies with negative/small effect sizes). The analysis is interpreted with caution due to the small number of included studies (*n* = 10).

Draw a funnel plot of the total effective rate. Most of the study points in the funnel plot are concentrated in the right area where the OR value is greater than 1, and their distribution is biased toward the upper half of the funnel outline. The funnel plot shows a certain degree of asymmetry, suggesting a potential risk of publication bias. However, due to the limited sample size, this result needs to be interpreted with caution. The reason may be that studies with negative results or small effect sizes have not been published. The results are shown in Figure 14.

## 4 Discussion

Diabetic nephropathy is an important complication of diabetes and is not only a major cause of chronic kidney disease worldwide (33). It is also closely related to cardiovascular diseases, the deterioration of kidney diseases, and mortality rates (34). Its characteristics include: ① Changes in kidney structure: such as thickening of the glomerular basement membrane, mesangial expansion, tubulointerstitial fibrosis, and glomerular hypertrophy; ② Impairment of kidney function: such as a decrease in glomerular filtration rate (GFR) and proteinuria (35). Its development can be divided into five stages: pre-nephropathy, asymptomatic stage, latent stage, overt nephropathy, and end-stage kidney disease (ESKD) (36). With the increase in the prevalence of diabetes, diabetic nephropathy imposes a huge economic and social burden on patients themselves and the healthcare system (37). Although existing treatment methods can reduce the risk of the onset and progression of diabetic nephropathy by strengthening blood glucose and blood pressure control, and inhibiting the renin-angiotensin system (RAAS) can also play a certain role in addressing the hemodynamic and metabolic abnormalities related to diabetic nephropathy, many patients still progress to end-stage renal disease (38). It requires treatment by dialysis or kidney transplantation. Given that the pathogenesis of diabetic nephropathy is multifactorial (39). Therefore, in addition to controlling blood sugar and blood pressure, exploring new treatment methods is particularly important (40). However, the complex pathophysiological mechanisms of diabetic nephropathy pose challenges to the development of effective therapeutic approaches (41). In addition, traditional Western medicine treatments for diabetic nephropathy may be accompanied by serious risks or adverse reactions (42). In recent years, adjuvant treatment methods based on traditional Chinese medicine have received widespread attention and achieved good results in the integration of traditional Chinese and Western medicine (43). The reason is that traditional Chinese medicine prescriptions and proprietary Chinese medicines usually contain a variety of medicinal materials, with multi-target and multi-level therapeutic effects, which can provide a material basis for the clinical efficacy of traditional Chinese medicine (44).

In ancient Chinese medical classics, although there is no term “diabetic nephropathy,” diabetic nephropathy can be categorized into the Chinese medicine concepts of “edema” and “consumptive thirst” based on its clinical symptoms, etiology, and pathogenesis (45). Diabetic nephropathy mainly involves the spleen and kidney, and its pathological nature is dominated by qi deficiency, yin deficiency, water retention, and blood stasis (46). Liuwei Dihuang Pills is a classic prescription first recorded in “Xiao'er Yaozheng Zhijue” (Guide to the Clinical Patterns of Pediatric Diseases) with a history of more than 1,000 years. It is widely used in the treatment of diabetic nephropathy. This medicine consists of six Chinese herbs: Rehmannia (prepared), Cornus officinalis, Moutan, Chinese yam, Poria, and Alisma. It was created by Qian Yi, a physician in the Song Dynasty, and is believed to have the effect of nourishing yin and tonifying the kidney (47). Modern research shows that Liuwei Dihuang Pills can reduce proteinuria in diabetic nephropathy, delay renal fibrosis, and protect renal mesangial cells by inhibiting signaling pathways such as NF-κB, MAPK, and TGF-β/SMADS (50).

This study systematically searched and included 11 eligible randomized controlled trials (RCTs), involving nearly 1,000 patients with early diabetic nephropathy. The clinical value of Liuwei Dihuang Pills (Decoction) combined with conventional western medicine treatment was evaluated through a rigorous meta-analysis.

In terms of the primary outcome, urinary albumin excretion rate (UAER), the experimental group showed a significant reduction compared with the control group, reflecting the potential role of Liuwei Dihuang Pills (Decoction) in reducing proteinuria and protecting the glomerular filtration membrane. egarding secondary outcomes, the experimental group also demonstrated significant advantages: among renal function-related indicators, the levels of serum creatinine (Scr) and blood urea nitrogen (BUN) were significantly lower than those in the control group, indicating that Liuwei Dihuang Pills (Decoction) may help reduce the filtration burden on the kidneys; at the same time, the mean arterial pressure of patients in the experimental group was controlled more stably, providing an important hemodynamic guarantee for renal function protection. Additionally, the total clinical effective rate of the experimental group was significantly higher than that of the control group. However, given that this indicator is based on the improvement of clinical symptoms, lacks standardized criteria, and was assessed without blinding in all included studies, the results should be interpreted with caution. The results of this study are consistent with the findings of the meta-analysis conducted by Lin et al. (48). This study included 18 randomized controlled trials and reported that Liuwei Dihuang Decoction combined with Western medicine significantly reduced levels of UAER, Scr, and BUN in patients with diabetic nephropathy. However, this study extends the existing evidence in several ways: First, it focuses on early-stage diabetic nephropathy, providing more targeted evidence for this key stage of the disease. Second, we conducted a detailed sensitivity analysis, identified high heterogeneity, and demonstrated that the overall conclusions remained robust after removing the main sources of heterogeneity. Third, we downgraded the overall quality of evidence to “low” according to the Grading of Recommendations Assessment, Development and Evaluation (GRADE) approach, reflecting the methodological limitations of the included studies, particularly the lack of allocation concealment and blinding (49). Fourth, we critically assessed the safety evidence and emphasized that only six studies reported adverse events, thus avoiding overly optimistic conclusions regarding safety.

In terms of adverse reactions, a total of 6 literatures reported relevant data. Chen et al. (23) reported that 4 cases of adverse reactions occurred in the experimental group, mainly manifested as fatigue, loss of appetite, nausea and vomiting, and skin rashes; 2 cases of adverse reactions occurred in the control group, mainly dizziness and fatigue. Li et al. (22) reported that 3 cases of adverse reactions occurred in the experimental group, including skin rashes, flushing, and dizziness, while 1 case of adverse reaction occurred in the control group, which was hypotension. Wang et al. (27) reported that 2 cases of adverse reactions occurred in the experimental group, mainly manifested as headache, facial flushing and ankle edema, and 3 cases of adverse reactions occurred in the control group with similar manifestations. Cao (29) It was reported that there was 1 case of hypoglycemia and 1 case of gastrointestinal reaction in the experimental group, and 4 cases of adverse reactions in the control group, mainly hypoglycemia and gastrointestinal reactions. No adverse reactions were observed in the remaining 2 studies. Overall, the adverse reactions reported in various studies were mostly mild to moderate, and could be relieved after symptomatic treatment. The meta-analysis showed no statistically significant difference in the incidence of adverse reactions between the two groups (OR = 0.99, 95% CI: 0.43 to 2.30, *P* = 0.99). However, given that only 6 of the 11 included studies reported adverse events, the safety evidence is subject to reporting bias, and the absence of reported adverse events in the remaining studies does not necessarily indicate their absence. Therefore, the safety findings should be interpreted with caution, and the current evidence is insufficient to definitively conclude that the combination therapy has a favorable safety profile.

## 5 Conclusion

Overall, Liuwei Dihuang Pills (Decoction) combined with conventional Western medicine may provide additional benefits for patients with early diabetic nephropathy, showing potential advantages in improving objective renal function indicators and clinical outcomes. Regarding safety, the available data suggest no significant increase in adverse events with the combination therapy; however, given that only 6 of the 11 included studies reported safety outcomes, the evidence is subject to reporting bias, and the absence of reported adverse events does not confirm their absence. Therefore, the safety conclusion remains uncertain and requires confirmation in future studies with standardized safety reporting.

This Meta-analysis still has several limitations that require further improvement in subsequent studies: (1) There is a limitation in the scope of included literature. This study only searched and included relevant Chinese-language literature, without incorporating English and other foreign-language literature. This may lead to selection bias due to language filtering, thereby restricting the external validity of the research results; (2) Methodological quality limitations: All included studies lacked allocation concealment and blinding, which may introduce selection and detection bias. Consequently, the overall evidence quality was downgraded to low according to GRADE principles; (3) The randomized controlled trials (RCTs) included generally have the problem of small sample sizes. Small-sample studies are susceptible to chance factors, which may reduce the statistical power of the results. In the future, there is an urgent need to conduct high-quality RCTs with large samples and multi-centers to enhance the reliability and persuasiveness of research data; (4) The clinical follow-up periods of the included studies are all relatively short, which makes it impossible to effectively evaluate the long-term efficacy and long-term medication safety of the combined treatment regimen with Liuwei Dihuang Pills (Decoction), and it is difficult to clarify its impact on the long-term progression of diabetic nephropathy; (5) There is a certain degree of heterogeneity among the included studies in terms of treatment regimens, medication doses, treatment courses, and patients' baseline data. This may interfere with the consistency of the Meta-analysis results and affect the accuracy of the conclusions. (6) Heterogeneity: Substantial heterogeneity was observed in several analyses (e.g., UAER, Scr), and although sensitivity analyses were performed, the high heterogeneity in some analyses limits the robustness of the pooled estimates. (7) Adverse events were only reported in 6 of the 11 included studies, and the remaining studies did not provide safety data. The incomplete reporting of adverse events introduces potential reporting bias and limits the reliability of the safety assessment. Therefore, the conclusion regarding safety remains uncertain and requires confirmation in future studies with standardized adverse event reporting.

Based on this, high-quality clinical studies with more scientific design, more comprehensive inclusion criteria, and longer follow-up periods should be conducted in the future. These studies will further verify the short-term and long-term efficacy and safety of Liuwei Dihuang Pills (Decoction) combined with conventional Western medicine in the treatment of early diabetic nephropathy. Meanwhile, in-depth exploration of its mechanism of action will provide a more solid evidence-based medicine basis for the standardized and individualized clinical application of this formula.

## Data Availability

The original contributions presented in the study are included in the article/supplementary material, further inquiries can be directed to the corresponding author.
